# Cut-Off Value for Pain Sensitivity Questionnaire in Predicting Surgical Success in Patients with Lumbar Disc Herniation

**DOI:** 10.1371/journal.pone.0160541

**Published:** 2016-08-05

**Authors:** Parisa Azimi, Edward C. Benzel

**Affiliations:** 1 Functional Neurosurgery Research Center, Shahid Beheshti University of Medical Sciences, Tehran, Iran; 2 Cleveland Clinic Foundation, Department of Neurosurgery, Cleveland, Ohio, United States of America; Universita degli Studi di Palermo, ITALY

## Abstract

Various factors related to predict surgical success were studied; however, a standard cut-off point for the Pain Sensitivity Questionnaire (PSQ) measure has not yet been established for a favorable surgical outcome for lumbar disc herniation (LDH). This study was to find the optimal cut-off point on the PSQ to distinguish surgical success in patients with LDH. A total of 154 patients with LDH consecutively referred to our clinic were enrolled into this prospective study between February 2011 and January 2014. All participants completed the PSQ. Patients completed the Oswestry Disability Index (ODI) score before surgery, and at 2 years after surgery. Surgical success was defined as a 13-point improvement from the baseline ODI scores. The cut-off value for PSQ was determined by the receiver-operating characteristic curve (ROC). The mean age of patients was 49.3±9.6 years, and there were 80 women. The mean time for follow-up assessment was 31±5 months (range 24–35). Post-surgical success was 79.9% (n = 123) at 2 years follow up. The mean score for the total PSQ, PSQ-minor, and PSQ-moderate were 6.0 (SD = 1.6), 5.4 (SD = 1.9) and 6.5 (SD = 1.7), respectively. Total PSQ score was also significantly correlated with the total scores of the ODI. The optimal total PSQ cut-off point was determined as > 5.2 to predict surgical success in LDH patients, with 80.0% sensitivity and 75.6% specificity (AUC-0.814, 95% CI 0.703–0.926). This study showed that the PSQ could be considered a parameter for predicting surgical success in patients with LDH, and can be useful in clinical practice.

## Introduction

Low back pain (LBP) has become one of the most serious public health problems [1]. Lumbar disc herniation (LDH) is one of the most common low back disorders associated with LBP. A herniated lumbar disc can press on the nerves in the spine and may cause pain, numbness, tingling or weakness in the foot [2]. Pain is a critical event in patients with spinal disorder that require attention from spine specialists [3]. Careful evaluation of each individual's pain sensitivity may become valuable for the prevention, assessment, and treatment of pain [3]. There are large individual differences in pain perception, and pain responsiveness. However, the Pain Sensitivity Questionnaire (PSQ) is predictive of pain-related responses to experimental stimuli. It has been developed as a simple and economical alternative to more time-consuming experimental testing methods that involve expensive equipment [4]. It has been validated as an outcome measure in LDH, [5] and in chronic pain and degenerative spinal disease [6–7]. In addition, the PSQ used to predict surgical outcomes for lumbar spinal stenosis [8].

As far as surgery for LDH is concerned, careful selection and screening for prognostic factors as pain sensitivity is crucial to minimize substantial costs and unfavorable outcomes. Hence, the question that remains is: does pain sensitivity decrease the success rate after surgery for LDH? In addition, what is a standard cut-off point for the PSQ measure in predicting surgical success in patients with lumbar disc herniation? Therefore, the aim of this prospective study was to determine an optimal cut-off point in order to establish a more accurate measure in predicting surgical success in patients with LDH. Previously we reported that the Predictive Score Card could predict surgical success in lumbar disc herniation [9], and here we report on the cut-off point for the Pain Sensitivity Questionnaire to build up additional knowledge on the topic. The data for patients in prior studies also were used in the present study. The former study was carried out from year 2010 to 2013 and the present study was carried out from February 2011 and January 2014. However, for both study we recruited 154 patients to fulfill the minimum sample size needed. The sample size was calculated based on 20% failure in surgical success.

## Methods

### Patients and data collection

A sample of newly diagnosed LDH patients referred to our hospital in Tehran, Iran consecutively enrolled into this prospective study. Data to assess the outcome for all patients who were to undergo discectomy with a single-level disc herniation were collected and patients were followed-up at least for 2 years. The diagnosis of LDH was made on the basis of clinical and radiographic evidence. All participants underwent a complete clinical examination for LDH including an assessment of clinical symptoms and clinical examination, and imaging studies—including plain radiography, computed tomography (CT) and magnetic resonance imaging (MRI) of the lumbar spine. In all cases, more than one spine surgeon confirmed the diagnosis, and surgery was performed by experienced surgeons. Patients were asked to fill out preoperative and follow-up questionnaires and to undergo follow-up examinations at last follow-up. There were no restrictions on patient selection with regard to types of LDH, age or other characteristics. Patients who had previous back surgeries and spinal anomalies were excluded.

Demographics including age, gender and Body-mass index (BMI), VAS associated with leg pain (mm) and VAS associated with back pain (mm) were determined. The duration of symptoms (in months), type of herniation and smoking histories were assessed. The time-point for postoperative assessment was 2 years after surgery.

### Operative Procedure

Standard open lumbar discectomy was used to manage LDH in patients who have persistent symptoms of the condition that do not improve with a conservative treatment [10].

### Measures

1. The Iranian version of the Pain Sensitivity Questionnaire (PSQ): Ruscheweyh et al. developed the Pain Sensitivity Questionnaire (PSQ) which assesses general pain sensitivity by self-rating without using extensive and painful experimental stimulation. This questionnaire consists of 17 questions, each describing a daily life situation on a numeric rating scale ranging from 0 (not painful at all) to 10 (worst pain imaginable). Of the 17 situations, most healthy subjects rated 14 items as painful. These painful situations involve a range of painful stimuli such as hot, cold, sharp, and blunt stimulation applied to different body parts including the head and upper and lower extremities. However, 3 of the 17 situations are normally not considering as painful by healthy subjects. These items are not included in the final score. The PSQ-total score could be calculated as the average rating of item 1, 2, 3, 4, 6, 7, 8, 10, 11, 12, 14, 15, 16, and 17 (all but the 3 non-painful items). The PSQ also has a minor score (PSQ-minor) and a moderate score (PSQ-moderate). The PSQ-minor that could be calculated as the average rating of 7 items (item 3, 6, 7, 10, 11, 12, and 14) and the PSQ-moderate score could be calculated as the average rating of 7 items (item 1, 2, 3, 8, 15, 16, and 17). The total PSQ score could be calculated as the mean of all items, excluding the 3 non-painful items. [S1 Appendix] [4–5].
2. The Iranian version of Oswestry Disability Index (ODI) (Version 2) was used to assess functionality. The ODI contains 10 items and its score range from 0 to 50, with higher scores indicating a worse condition. The psychometric properties of the Iranian version of questionnaire are well documented [11]. The ODI score was measured at admission and at last follow-up. A minimum clinically important difference (MCID) is a threshold used to calculate the effect of clinical treatments. Surgical success was defined as a 13-point improvement from the baseline ODI scores [12].
3. The Finneson–Cooper score was also used. This is a lumbar disc surgery predictive score card or questionnaire that was developed by Finneson–Cooper to assess potential candidates for excision of a herniated lumbar disc [13]. The Finneson–Cooper score range from 0 to 100 and it categorizes candidates into a 4-grade classification: good >75; fair 65–75; marginal 55–64, and poor < 55.

### Statistical analysis

The total PSQ score used for the optimal cut-off. Sensitivity and specificity calculations and receiver operating characteristic (ROC) analysis was used to estimate the best cut-off points for PSQ to predict surgical success [14]. Areas under the curve (AUC) were calculated as measures of the accuracy of the tests. The AUC is a measure of discrimination; a model with a high area under the ROC curve suggests that the model is able to accurately predict the rate of an observation’s response. The AUC was interpreted as follows: no discrimination (AUC = 0.50), acceptable discrimination (0.7≤ AUC <0.8), excellent discrimination (0.8≤ AUC <0.9), and outstanding discrimination (AUC more than 0.9) [15]. In addition, the correlation between the PSQ and the ODI was assessed using Pearson’s correlation coefficient in preoperative. All statistical analyses were performed using the PASW Statistics 18 Version 18 (SPSS, Inc., 2009, Chicago, IL, USA) and P < 0.05 was considered to indicate a statistically significant difference.

### Ethics

Each participant gave informed verbal consent. Since some patients were less educated, for consistency we only asked for verbal consent. The main investigator explained the study for each participant and asked for permission. It was indicated that participation and no participation does not influence the treatment and their information will remain confidential. The Ethics Committee of Shahid Beheshti University of Medical Sciences, Tehran, Iran, approved the study and agreed with the consent procedure.

## Results

Of the original 177 patients, 154 (74 men and 80 women) were included in the study; 13 were excluded because of deficient follow-up results, three patients due to recurrent disk herniations, and 7 cases due to spinal anomalies. The demographics of the LDH patients and their scores on the Finneson–Cooper score, the PSQ, and the ODI are shown in Table 1. Mean total PSQ, PSQ minor, and PSQ-moderate were observed 6.0 (SD = 1.6), 5.4 (SD = 1.9) and 6.5 (SD = 1.7), respectively. The total PSQ was ranging from 2.8 to 9.9.

**Table 1:** Demographic data and preoperative status of patients with lumbar disc herniation (n = n = 154).

Characteristics	Mean (SD)
Age (Year)	49.3 (9.6)
*Range*	21–80
Gender (Male; n, %)	74 (48.1)
Smoking (n, %)	57(37.0)
Body-mass index (BMI)	25.4 (4.8)
**Symptoms**	
Duration of symptoms (months)	16.1(12.1)
*Range*	1–25
VAS of leg pain (mm)	59.1 (18.6)
*Range*	17–100
VAS of back pain (mm)	54.9(24.4)
*Range*	19–100
**ODI**	
Baseline	38.6 (14.3)
At last follow-up	16.4 (11.3)
Satisfied (n, %)	123 (79.9)
Dissatisfied (n, %)	31 (20.1)
**PSQ** **	
PSQ -minor (0–10)	5.4 (1.9)
PSQ -moderate (0–10)	6.5 (1.7)
PSQ–total (0–10)	6.0 (1.6)
**Finneson–Cooper score (n, %)**	
Good	101 (65.6)
Fair	53 (34.4)
**Level of hearniation (n, %)**	
L1-L2	3 (1.9)
L2-L3	6 (3.9)
L3-L4	19 (12.3)
L4-L5	76 (49.4)
L5-S1	50 (32.5)
**Type of herniation (n, %)**	
Sequestration	46 (29.9)
Transligamentous extrusion	58 (37.7)
Subligamentous extrusion	37 (24.0)
Protrusion	13 (8.4)

Values are mean (SD), number or percentage

** Lower scores on the PSQ indicate better conditions.

Based on the ODI, post-surgical success was 79.9% (n = 123). Mean improvement in the ODI was 22.2±12.4 and statistically was significant (p<0.001) at 2-year follow-up. No significant differences were observed for post-surgical success between levels of LDH. Total PSQ were also significantly correlated with the total scores of the ODI (p <0.01). The total PSQ score by Finneson–Cooper score for Good (Finneson–Cooper score >75) and Fair (Finneson–Cooper score 65–75) were 6.8 (SD = 1.7) and 4.5 (SD = 1.3), respectively. Patients with a Finneson-Cooper ‘‘good” grade would achieve a greater increase in the PSQ score compared with cases with a Finneson-Cooper ‘‘fair” grade.

**Fig 1:**
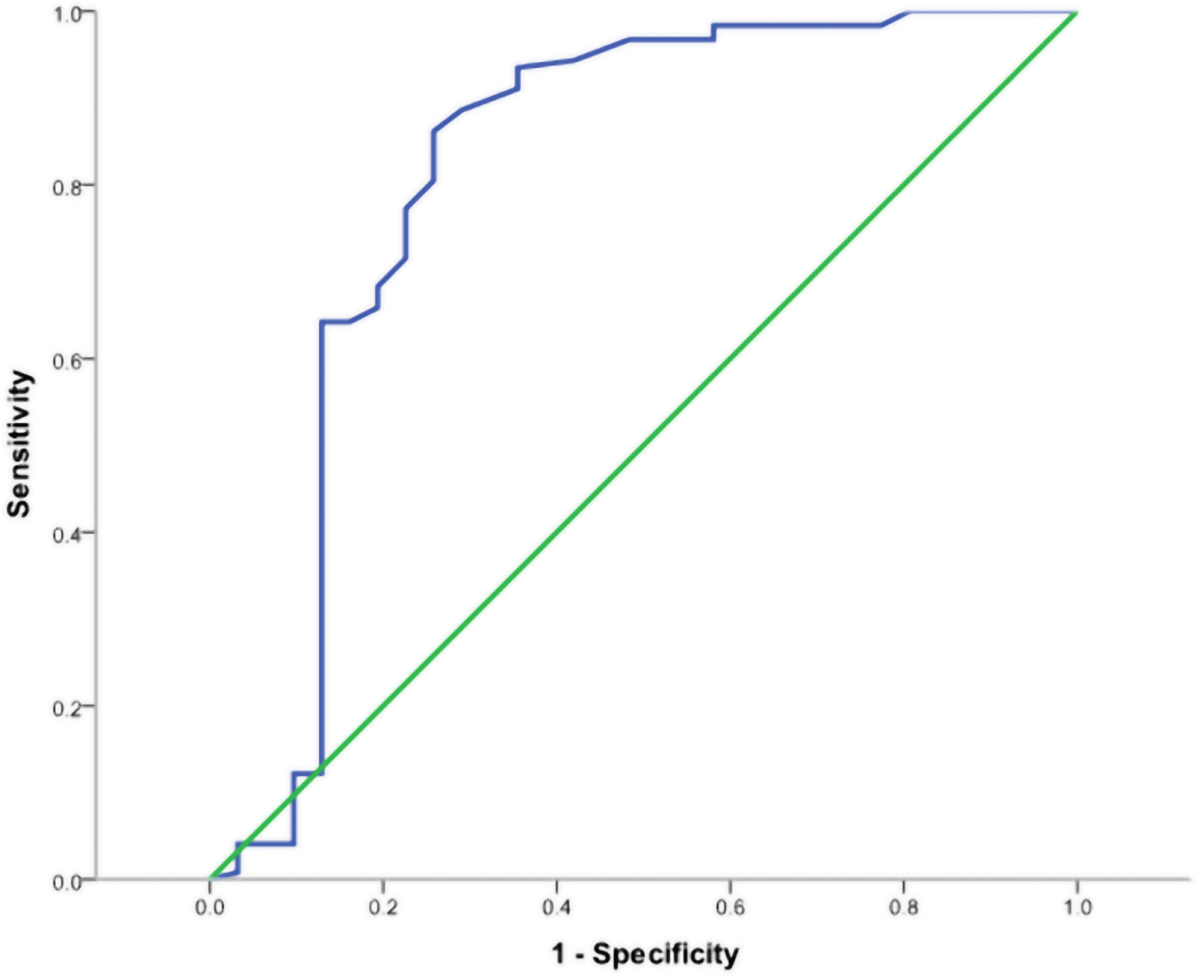
ROC Curve for the total PSQ as a predictor of surgical success in patients with LDH (AUC-0.814, 95% CI 0.703–0.926; P < 0.001). The optimal cut-off for maximum sensitivity and specificity was > 5.2.

According to the ROC analysis, the optimal cut-off value of PSQ to predict surgical success was measured as more than 5.2, with 80.0% sensitivity and 75.6% specificity (AUC-0.814, 95% CI 0.703–0.926). The finding is shown in Fig 1.

No discectomy case was observed to have a missed level surgery. Cauda-equina syndrome occurred in two cases (1.3%). In one case (0.65%) dural laceration occurred during surgery which were repaired and no one showed CSF leakage or meningitis. No mortality rate was observed due to surgery.

## Discussion

The present study aimed to determine the optimal cut-off point for the PSQ to predict surgical success in patients with LDH, and the proposed optimal cut-off value was > 5.2, based on the point producing the greatest discriminatory ability on ROC analysis. In the other words a PSQ of > 5.2 is a predictor of success in LDH surgery.

Few high quality studies were found in which back pain and leg pain independently predicted surgical success in patients with LDH [16–18]. Overall, pain was always associated with LDH post-operative outcomes. Moreover, back pain and leg pain seemed to have different prognostic values. Patients with greater baseline back pain were associated with worse outcomes [16–17]. On the other hand, patients with higher baseline leg pain had better surgical outcomes [16, 18]. Therefore, the present results are in line with those of previous studies of pain sensitivity. This was also true for other spine studies [19–22].

In this paper, a PSQ cut-off value was determined to predict surgical success in patients with LDH by ROC analysis; to our knowledge, this is the first attempt to determine the cut-off value in this manner. We believe that this is a logical and reasonable way to define the cut-off value for PSQ. Another report had determined PSQ cut‐off point to predict surgical success in patients with lumbar spinal stenosis (LSS) by ROC analysis in other ethnic population [8]. ROC analysis revealed AUCs for the total PSQ score of 0.638 (95% CI = 0.546–0.729, P = 0.005). The possible reasons for the inconsistency might be related to the differences in type of disease, ethnicity and clinical backgrounds, including body mass index. Moreover, the present result shows higher sensitivity and specificity than the value of the other study.

The Finneson–Cooper score as a clinical measure for known-groups comparison. The findings showed that patients who differed in Finneson–Cooper score assessments scored differently on the PSQ score. In addition, total PSQ were also significantly correlated with the total scores of the ODI. In fact such a result lends support to the discriminant validity of the PSQ score.

This study has some important limitations. First, our study suggests that a PSQ level cut-off value of > 5.2 can be used to detect surgical success in patients with LDH. However, our findings do not imply that one cannot use cut-off values other than >5.2. In this case, practitioners or researchers may have to make a trade-off when choosing the cut-off value. A cut-off value of > 5.2 could be chosen if practitioners or researchers are ready to accept more false positives. Second, this is a cut‐off point in patients with LDH in an Iranian population. So the cut‐off value specific for the other population is needed. Third, a gender difference was not assessed. However, Tschugg et al. reported that gender differences in pain perception not only exist in healthy subjects, but also in patients with LDH [23]. Henceforth, further studies are needed to evaluate this issue. Fourth, due to the lack of a true gold standard for assessing the patient surgical success, certain cases may have been incorrectly classified. Hence, a standardized method for assessment of successful outcome is needed. Fifth, the impact of rehabilitation treatments on the last outcome of the patients should be assessed. Finally, more sophisticated multivariate statistical analyses should have been performed to determine whether there was any association between PSQ and age, gender, smoking status, baseline VAS, baseline ODI, etc.

## Conclusion

Although follow-up studies are required to confirm the feasibility of the definition of surgical success in patients with LDH, the cut-off value of the PSQ as a criterion for patients with LDH in Iran should be > 5.2 based on the present results.

## Supporting Information

S1 Appendix Pain Sensitivity Questionnaire. (DOC)
