# Supplementary material for: Cut-Off Value for Pain Sensitivity Questionnaire in Predicting Surgical Success in Patients with Lumbar Disc Herniation
Source: PLoS One. 2016 Aug 5;11(8):e0160541. doi: 10.1371/journal.pone.0160541 (PMC4975474; doi:10.1371/journal.pone.0160541)
Supplement: S1 Appendix — (DOC) [file pone.0160541.s001.doc]

**Appendix1:** Pain Sensitivity Questionnaire

| **Pain Sensitivity Questionnaire** | |
| --- | --- |
| **Pain sensitivity-minor** | 3. Imagine your muscles are slightly sore as the result of physical activity |
|  | 6. Imagine you have mild sunburn on your shoulders. |
|  | 7. Imagine you grazed your knee falling off your bicycle |
|  | 10. Imagine you have a minor cut on your finger and inadvertently get lemon juice in the wound |
|  | 11. Imagine you prick your fingertip on the thorn of a rose. |
|  | 12. Imagine you stick your bare hands in the snow for a couple of minutes or bring your hands in contact with snow for some time, for example, while making snowballs |
|  | 14. Imagine you shake hands with someone who has a very strong grip |
| **Pain sensitivity-moderate** | 1. Imagine you bump your shin badly on a hard edge, for example, on the edge of a glass coffee table |
|  | 2. Imagine you burn your tongue on a very hot drink |
|  | 4. Imagine you trap your finger in a drawer |
|  | 8. Imagine you accidentally bite your tongue or cheek badly while eating |
|  | 15. Imagine you pick up a hot pot by inadvertently grabbing its equally hot handles |
|  | 16. Imagine you are wearing sandals and someone with heavy boots steps on your foot |
|  | 17. Imagine you bump your elbow on the edge of a table (“funny bone”) |
